# Attraction Controls the Entropy of Fluctuations in Isosceles Triangular Networks

**DOI:** 10.3390/e20020122

**Published:** 2018-02-12

**Authors:** Fabio Leoni, Yair Shokef

**Affiliations:** 1 School of Mechanical Engineering, Tel-Aviv University, Tel-Aviv 69978, Israel; 2 Sackler Center for Computational Molecular and Materials Science, Tel-Aviv University, Tel-Aviv 69978, Israel

**Keywords:** order by disorder, geometric frustration, confined colloids, residual entropy

## Abstract

We study two-dimensional triangular-network models, which have degenerate ground states composed of straight or randomly-zigzagging stripes and thus sub-extensive residual entropy. We show that attraction is responsible for the inversion of the stable phase by changing the entropy of fluctuations around the ground-state configurations. By using a real-space shell-expansion method, we compute the exact expression of the entropy for harmonic interactions, while for repulsive harmonic interactions we obtain the entropy arising from a limited subset of the system by numerical integration. We compare these results with a three-dimensional triangular-network model, which shows the same attraction-mediated selection mechanism of the stable phase, and conclude that this effect is general with respect to the dimensionality of the system.

## 1. Introduction

Geometrically-constrained systems may show peculiar features compared to their unconstrained counterparts. In particular, geometric constraints can lead to frustration because the system cannot simultaneously minimize all local interaction energies, or free energies. Frustrated systems usually show a degenerate ground state and then they may posses a residual entropy. Frustration is relevant in physical and biological systems that range from water [1] and spin ice [2,3] to magnets [4], magnetic island [5], high transition-temperature superconductors [6], elastic beams [7,8] and colloids [9,10,11,12]. The possibility to control colloidal interparticle interactions and to visualize and manipulate each particle and follow its motion in both space and time makes colloidal suspension a powerful tool to study phenomena in condensed-matter physics, ranging from glass formers [13], to crystals and gels [14].

**Figure 1:**
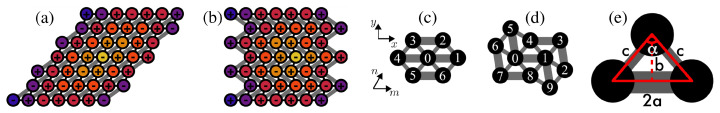
(**a**) Straight and (**b**) maximally zigzagging or bent configuration. Shells, which order is indicated with $n_{s}$, are denoted by increasingly darker colors for increasing $n_{s}$. Thicker, gray lines correspond to springs of longer (for α>π/3) or shorter (for α<π/3) length at rest; (**c**,**d**) are the unit cell for the straight- and bent-stripes confgirations, respectively. Numbers associated to particles correspond to the particle positions in tables in Section 2; (**e**) Parameters associated to every plaquette: *a*, *b*, *c* and α.

A prototypical geometrically-confined system is composed of short-range repulsive colloids confined in a slit pore of two plates. Varying density and plate separation, discontinuous phase transitions between layered, buckled, rhombic and adaptive prism crystal structures occur [15,16,17]. In the case of a slit pore with a plates interdistance slightly larger than a colloid diameter, when density approaches the close-packing value $\rho_{cp}$, colloids, due to their free-volume-dominated free energy, tend to touch opposite walls, giving rise to effective antiferromagnetic interactions [9,18], and to glassy dynamics [19]. Multiple configurations corresponding to the same $\rho_{cp}$ can be obtained by alternating straight stripes of up and down spheres (Figure 1a) or by any set of zigzagging stripes (Figure 1b).

This ground-state degeneracy implies a subextensive residual entropy at $\rho_{cp}$ ($S_{0}∼\sqrt{N}$, with *N* the number of particles in the system) [9] so that the residual entropy per particle tends to zero in the thermodynamic limit. At $\rho =\rho_{cp}$, in the straight- or zigzagging-stripes configuration, a colloidal sphere is surrounded by two colloids touching the same wall (giving rise to frustrated bonds) and by four colloids touching the opposite wall (satisfied bonds), except for the limit case of $\alpha =90^{\circ }$, in which case every sphere is surrounded by four colloids touching the same wall and four colloids touching the opposite wall, where α is defined in Ref. [20]. The terms frustrated and satisfied bonds refer both to the packing consideration of neighboring spheres wanting to touch opposite walls, and also from the obvious connection to an antiferromagnetic spin-1/2 Ising model [18]. In this case, nearest neighbors at opposite spin states satisfy the antiferromagnetic interaction between them, while those at the same spin state represent the frustration of this Ising model on the triangular lattice [21]. The three-dimensional (3D) network of links connecting the centers of neighboring spheres is composed of tilted equilateral triangles, since the 3D distance between the centers of each pair of contacting spheres is equal to the sphere diameter. When the centers of the colloids are projected on the plane parallel to the plates, it reduces to a 2D network composed of isosceles triangles. In this network, the longer link of each triangular plaquette corresponds to a frustrated bond, while the two shorter links correspond to satisfied bonds. Straight and zigzagging stripes are the only configurations corresponding to a 2D network of isosceles triangles which can tile the plane [9].

This geometric mechanism underlying the ground state of the buckled colloidal system composed of straight or zigzagging stripes, has been realised, experimentally also by packing a granular system in a container under the effect of gravity [22,23], and theoretically with spins starting from the Wannier antiferromagnetic Ising model on a triangular lattice [21] by allowing for the lattice to elastically deform [24,25]. Zigzagging stripes patterns have been found also in the Ising model on an anisotropic triangular lattice [26]. A relevant phenomenon involving the selection of one between two competing phases related to the distorsion of equilateral into iscosceles triangles and due to angular frustrations concerns stiff, nematically ordered, polymer molecules such as DNA [27]. The Ising antiferromagnet on a deformable triangular lattice has the same degeneracy of the ground state and subextensive entropy at T=0 as the colloidal system at $\rho =\rho_{cp}$ (that is $S_{0}∼\sqrt{N}$, equivalent to the $N^{2/3}$ scaling found in Perovskite Oxynitrides [28]). In the following we refer to T=0 as temperature being arbitrarily close to zero. Indeed, it has been shown that the third law of thermodynamics, implying the unattainability of absolute zero temperature in a finite number of steps and within a finite time, holds for arbitrary classical or quantum systems or involving infinite-dimensional reservoirs [29].

For temperature slightly larger than zero, the degeneracy is removed by the order-by-disorder effect [30,31,32,33,34,35,36,37] and in the elastic Ising model straight stripes represent the stable phase [20,25], while bent stripes are selected in the colloidal system for $\rho <\rho_{cp}$ when colloids are modeled using hard or soft repulsive potentials [20]. By tuning the attractive vs repulsive components of an asymmetric power-law potential used to model colloids, we found that the stable phase in the colloidal system can be turned from bent to straight stripes for attraction strong enough compared to repulsion [20]. We established a connection between the effect of the attraction on the phase stability and the packing of hard spheres and their entropy. We showed that other parameters of these systems are irrelevant to the phase stability, as for example their dimensionalities. Indeed, the elastic Ising antiferromagnet is defined in 2D, while the colloidal system is 3D or quasi-2D due to the buckling of the monolayer.

In this paper we study a 2D isosceles triangular network which shows the same ground-state degeneracy as the Ising elastic antiferromagnet at T=0 or the colloidal monolayer at $\rho =\rho_{cp}$. Increasing temperature above zero, the degeneracy is removed through the order-by-disorder effect and we find the straight-stripes phase to be selected for particles linked with harmonic interactions, while the bent-stripes phase is more stable if only the repulsive component of the harmonic inter-particle potential is considerd (that we call *repulsive harmonic potential*, described in detail in the following section). This result suggests that the inversion of the stable phase by adding attraction to repulsively-interacting particles in triangular networks is a general mechanism, irrespective of the dimensionality of the system.

## 2. Isosceles Triangular Network Model

The model we consider is composed of particles in a 2D triangular network linked with springs of two different lengths at rest such that every plaquette or triangle is formed by a longer edge 2a and two shorter identical edges *c*, and thus has a height $b=\sqrt{c^{2}-a^{2}}$ and a head angle α such that a=btan(α/2) (see Figure 1e). We will first consider particles interacting through a harmonic potential and then will consider a repulsive harmonic potential, as defined below. The Hamiltonian of the system for harmonic inter-particle interaction can be written as

(1) $$H^{h}=\sum_{m,n}\sum_{l=1}^{3}\frac{K}{2}{\left(dr_{l}-dr_{0}\right)}^{2},$$

where *K* is the spring constant, which is assumed to be identical for all springs, 1≤m,n≤L, $N=L^{2}$ is the number of particles or nodes of the network and the index *l* runs over three of the six neighbors each particle has to avoid double counting of bonds. The positions of the particles are described by the coordinates $\left\{x_{i},y_{i}\right\}$. At T=0 multiple degenerate states, represented by straight or zigzagging stripes, minimize the system free energy. For T>0 the system cannot jump from one configuration to another, but at T=0 it is at mechanical equilibrium in every state under consideration. Therefore, the present model is not ergodic by construction. In the conclusion we will discuss entropy calculation in non ergodic systems. In order to study the stability of the system at T>0, we will consider small fluctuations about the equilibrium position described by small displacements $\left\{u_{i},v_{i}\right\}$ of all particles in the straight and bent configurations. dr is the distance between particles *i* and *j*, and its square is thus given by

(2) $$dr^{2}={\left(dx+du\right)}^{2}+{\left(dy+dv\right)}^{2}=dr_{0}^{2}+2\left(dxdu+dydv\right)+du^{2}+dv^{2}$$

where $dx=x_{i}-x_{j}$, $dy=y_{i}-y_{j}$, $du=u_{i}-u_{j}$, $dv=v_{i}-v_{j}$ and $dr_{0}={\left(dx^{2}+dy^{2}\right)}^{1/2}$ is the length at rest of the spring linking particles *i* and *j*, which can take the values *c* or 2a (see Figure 1e, Table 1 and Table 2). Since we consider the expansion around mechanical equilibrium, we will drop terms linear in du and dv and write: $dr^{2}=dr_{0}^{2}+du^{2}+dv^{2}$. Expanding to harmonic order the expression of dr that we get from Equation (2), we obtain

(3) $$dr=dr_{0}+\frac{du^{2}}{2dr_{0}}\left(1-\frac{dx^{2}}{dr_{0}^{2}}\right)+\frac{dv^{2}}{2dr_{0}}\left(1-\frac{dy^{2}}{dr_{0}^{2}}\right)-\frac{dxdydudv}{dr_{0}^{3}}.$$

The Hamiltonian of the straight-stripes configuration, with particle positions specified for the unit-cell in Table 1, is

(4) $$H_{s}^{h}=\frac{K}{2}\sum_{m,n}\left[du_{1}^{2}+\frac{a^{2}}{c^{2}}\left(du_{2}^{2}+du_{3}^{2}\right)+\frac{b^{2}}{c^{2}}\left(dv_{2}^{2}+dv_{3}^{2}\right)-\frac{2ab}{c^{2}}\left(-du_{2}dv_{2}+du_{3}dv_{3}\right)\right].$$

Using the relations $du_{l}=u_{l}-u_{0}$ and $dv_{l}=v_{l}-v_{0}$ we get

(5) $$\begin{array}{cc} H_{s}^{h}= & K\sum_{m,n}\left[u_{0}^{2}-u_{0}u_{1}+\frac{a^{2}}{c^{2}}\left(2u_{0}^{2}-u_{0}u_{2}-u_{0}u_{3}\right)+\frac{b^{2}}{c^{2}}\left(2v_{0}^{2}-v_{0}v_{2}-v_{0}v_{3}\right)\right.\\ & \left.-\frac{2ab}{c^{2}}\left(u_{0}v_{2}+u_{2}v_{0}-u_{0}v_{3}-u_{3}v_{0}\right)\right]. \end{array}$$

The Hamiltonian of the bent-stripes configuration, with particle positions specified for the unit-cell in Table 2, is

(6) $$\begin{array}{cc} H_{b}^{h}= & \frac{K}{2}\sum_{t,n}\left\{du_{10}^{2}+\cos^{2}\alpha du_{50}^{2}+\sin^{2}\alpha dv_{50}^{2}-\sin\left(2\alpha \right)du_{50}dv_{50}+\sin^{2}\left(\frac{\alpha }{2}\right)\left(du_{40}^{2}+du_{41}^{2}+du_{31}^{2}\right)\right.\\ & +\sin^{2}\left(\frac{3\alpha }{2}\right)du_{21}^{2}+\cos^{2}\left(\frac{\alpha }{2}\right)\left(dv_{40}^{2}+dv_{41}^{2}+dv_{31}^{2}\right)+\cos^{2}\left(\frac{3\alpha }{2}\right)dv_{21}^{2}\\ & \left.-\sin\alpha \left(-du_{40}dv_{40}+du_{41}dv_{41}-du_{31}dv_{31}\right)+\sin\left(3\alpha \right)du_{21}dv_{21}\right\}, \end{array}$$

where 1≤t≤L/2. Indeed, the unit-cell of the bent stripe configuration includes two particles (see Figure 1d): particle 0, which represents the particles with odd *m*, and particle 1, which represents the particles with even m. Therefore, we set for particle 0, m=2t−1, and for particle 1, m=2t. Using the relations $du_{l0}=u_{l}-u_{0}$, $du_{l1}=u_{l}-u_{1}$ and $dv_{l0}=v_{l}-v_{0}$, $dv_{l1}=v_{l}-v_{1}$ we get

(7) $$\begin{array}{cc} H_{b}^{h}= & \frac{K}{2}\sum_{t,n}\left\{\left[2-\sin^{2}\alpha +3\sin^{2}\left(\frac{\alpha }{2}\right)+\sin^{2}\left(\frac{3\alpha }{2}\right)\right]\left(u_{0}^{2}+u_{1}^{2}\right)+\left(\sin\alpha -\sin2\alpha +\sin3\alpha \right)(u_{0}v_{5}\right.\\ & +u_{5}v_{0})-\sin\alpha \left(u_{0}v_{4}+u_{4}v_{0}-u_{1}v_{4}-u_{4}v_{1}+u_{1}v_{3}+u_{3}v_{1}\right)-\sin3\alpha \left(u_{1}v_{2}+u_{2}v_{1}\right)\\ & +\left[1-\cos^{2}\alpha +3\cos^{2}\left(\frac{\alpha }{2}\right)+\cos^{2}\left(\frac{3\alpha }{2}\right)\right]\left(v_{0}^{2}+v_{1}^{2}\right)-2\sin^{2}\alpha v_{0}v_{5}-2\cos^{2}\left(\frac{\alpha }{2}\right)(v_{0}v_{4}+v_{1}v_{3}\\ & \left.+v_{1}v_{4})-2\cos^{2}\left(\frac{3\alpha }{2}\right)v_{1}v_{2}\right\}. \end{array}$$

**Figure 2:**
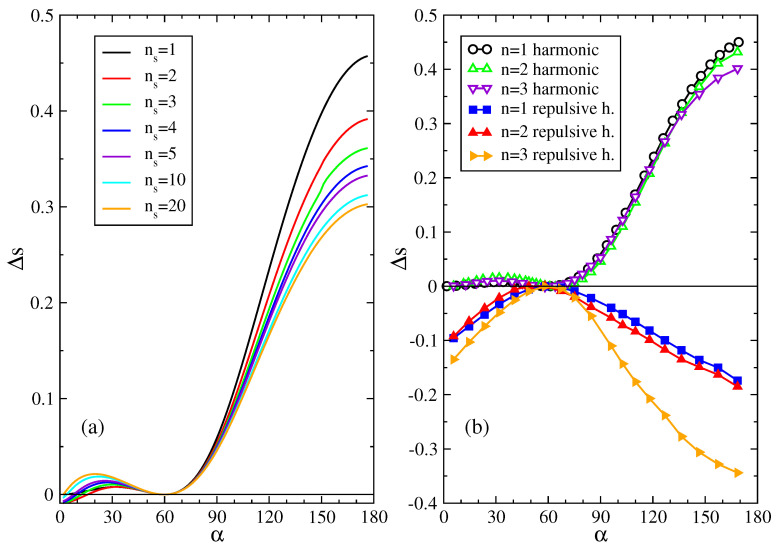
(**a**) Entropy difference per particle Δs, between straight- and bent-stripe configurations vs. deformation angle α for increasing numbers of shells $n_{s}$ of fluctuating particles; (**b**) Δs vs. α for n=1,2,3 particles free to move for harmonic (open symbols) and repulsive harmonic (filled symbols) interactions.

For a harmonic interparticle potential, the Hamiltonian for straight and bent stripes configurations we expanded around mechanical equilibrium takes the quadratic form: $H=K\sum_{m,n}A_{m,n}q_{m}q_{n}$, where {q}={u,v} represents small displacements about the equilibrium position of every particle. In the canonical ensemble the difference between the entropy per particle of the straight and bent configurations for such Hamiltonian is [20]: $\Delta s=\left(S_{s}-S_{b}\right)/N=1/\left(2N\right)\ln(∥A_{b}∥/∥A_{s}∥)$ where the subscript *s* refers to straight and *b* to bent, and ∥A∥ is the determinant of A. The dimensionless matrix A depends only on the deformation angle α and on the zigzagging-stripe realization. In [20] we used a recursive method to obtain the matrix A in the case of the elastic Ising model for any subset of the network composed by shells of particles (see Figure 1). Here we apply the same method to the 2D spring network model. The number of particles *n* belonging to the shells up to $n_{s}$ is given by $n=1+3n_{s}\left(n_{s}-1\right)$. In our shell-expansion calculation, these *n* particles are free to move, while the other N−n particles of the network are frozen in their equilibrium position. Increasing *n*, Δs should converge to the exact result (see Figure 2a), which includes the simultaneous fluctuation of all particles in the system.

In Figure 2a we show Δs for the 2D harmonic network model for a number of shells up to $n_{s}=20$, that is n=1141 particles free to move. From it we can see that Δs>0 for every deformation angle α of the network and for every order $n_{s}$ of the expansion (except for very small deviations at small α and small $n_{s}$). Considering only one particle free to move, $n_{s}=1$, gives a qualitative indication on the behavior of Δs for every orders of approximation. This rapid convergence with increasing $n_{s}$ gives confidence in this expansion method when we will apply it for purely repulsive interactions, for which we are technically much more limited in the number of particles that we may numerically calculate the simultaneous fluctuation of.

Now we consider the same 2D triangular network model, but for repulsive harmonic interactions, that is we consider the following Hamiltonian

(8) $$H^{r}=\sum_{m,n}\sum_{l=1}^{3}\frac{K}{2}{\left(dr_{l}-dr_{0}\right)}^{2}\theta \left(dr_{0}-dr_{l}\right),$$

where θ() is the Heaviside step function. Namely, now each spring applies a restoring force only when compressed ($dr_{l}<dr_{0}$) and there is no resistance to stretching ($dr_{l}>dr_{0}$). In this case we have to numerically integrate the partition function in order to get the entropy of straight- and bent-stripe configurations. In Figure 2b we show Δs for repulsive harmonic interactions for n=1,2,3. Numerical calculations for n>3 are beyond our computational reach (numerical integration for a number of variables larger than 6, in our case suffers from fluctuating results for a limited capacity in the precision). For n=1 particle free to move, each particle can be equivalently chosen to be free. For n=2 we consider the particles 0 and 1 (see Figure 1c,d). For n=3 we consider the particles 0, 1, 2 and 0, 1, 4 for the straight and bent configurations, respectively (see Figure 1c,d). As for the 3D spring network of [20], we find that also in our 2D model Δs<0 for repulsive interactions.

For the case n=1 we can show how the inversion of the stable phase when turning from harmonic to repulsive harmonic potential depends on the spatial configurations of straight and bent stripes, and in particular of the angular distribution of neighboring particles around each particle in the network.

**Figure 3:**
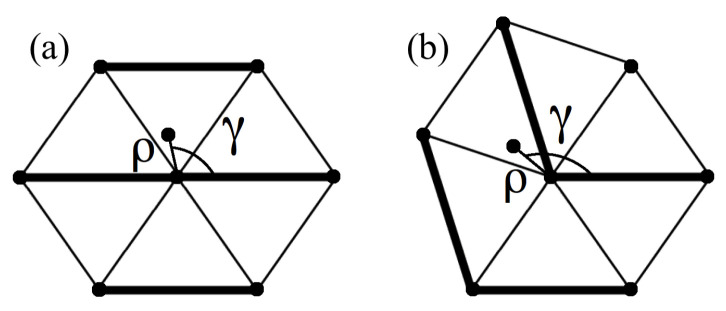
Example of straight (**a**) and bent (**b**) configuration for n=1 particle free to move. The deviation of the central particle from its equilibrium position is described by polar coordinates (ρ,γ).

For n=1 the computation of the canonical partition function for the repulsive harmonic system can be easily reduced to the integration of single variable functions using polar coordinates (ρ,γ) (see Figure 3). The Hamiltonian of the free particle 0 can be written as $H_{0}^{r}=1/2K\rho^{2}\sum_{i=1}^{6}g_{i}\left(\alpha ,\gamma \right)$ where $\rho^{2}g_{i}\left(\alpha ,\gamma \right)$ is the contribution to $H_{0}^{r}$ coming from the neighbor *i* of the particle 0, and the function $g_{i}$ depends on the coordinates of the particle *i*, as specified below. The canonical partition function is thus

(9) $$Z_{0}^{r}=\int_{0}^{\infty }\int_{0}^{2\pi }\exp\left[-\beta \frac{K}{2}\rho^{2}\sum_{i=1}^{6}g_{i}\left(\alpha ,\gamma \right)\right]\rho d\rho d\gamma =\frac{1}{\beta K}\int_{0}^{2\pi }\frac{d\gamma }{\sum_{i=1}^{6}g_{i}\left(\alpha ,\gamma \right)}=\frac{I(\alpha )}{\beta K}$$

where $\beta =1/(K_{B}T)$ is the Boltzmann factor and $I\left(\alpha \right)=\int_{0}^{2\pi }{\left[\sum_{i=1}^{6}g_{i}\left(\alpha ,\gamma \right)\right]}^{-1}d\gamma =\int_{0}^{2\pi }f^{-1}\left(\alpha ,\gamma \right)d\gamma$ with $f\left(\alpha ,\gamma \right)=\sum_{i=1}^{6}g_{i}\left(\alpha ,\gamma \right)$. In this case we have $\Delta s=\ln(I_{s}\left(\alpha \right)/I_{b}\left(\alpha \right))$.

The difference between the calculation of the harmonic and the repulsive harmonic partition function is that in the former case the functions $g_{i}$ contributes to the integral I(α) for any angle 0≤γ≤2π, while in the latter case every $g_{i}$ contributes to I(α) for a specific range of angles only. For the repulsive harmonic potential in the straight-stripes configuration we need to consider the azimuthal ranges coming from each one of the neighboring particles:

(10) $$\begin{array}{cc} f_{s}^{r}\left(\alpha ,\gamma \right)= & \cos^{2}\gamma \left[\theta (\gamma )\theta (\pi /2-\gamma )+\theta (\gamma -3\pi /2)\theta (2\pi -\gamma )\right]+\sin^{2}\left(\alpha /2+\gamma \right)[\theta \left(\gamma \right)\theta \left(\pi -\alpha /2-\gamma \right)\\ & +\theta \left(\gamma -2\pi +\alpha /2\right)\theta \left(2\pi -\gamma \right)]+\sin^{2}\left(\alpha /2-\gamma \right)\left[\theta (\gamma -\alpha /2)\theta (\pi +\alpha /2-\gamma )\right]\\ & +\cos^{2}\gamma \left[\theta (\gamma -\pi /2)\theta (3\pi /2-\gamma )\right]+\sin^{2}\left(\alpha /2+\gamma \right)\left[\theta (\gamma -\pi +\alpha /2)\theta (2\pi -\alpha /2-\gamma )\right]\\ & +\sin^{2}\left(\alpha /2-\gamma \right)\left[\theta (\gamma -\pi -\alpha /2)\theta (2\pi -\gamma )+\theta (\gamma )\theta (\alpha /2-\gamma )\right]. \end{array}$$

Due to the symmetry of the straight-stripes configuration, thanks to which the reflection about the origin of each neighbor transforms it in another neighboring particle (see Figure 1c and Table 1), we can write the function $f_{s}^{r}$ by just taking the contribution of every $g_{i}$ without the condition imposed by the Heaviside step functions and dividing it by 2, that is

(11) $$f_{s}^{r}\left(\alpha ,\gamma \right)=\cos^{2}\gamma +\sin^{2}\left(\alpha /2+\gamma \right)+\sin^{2}\left(\alpha /2-\gamma \right)=\cos^{2}\gamma \left(2-\cos\alpha \right)+\cos^{2}\left(\alpha /2\right)$$

For the repulsive harmonic potential in the bent configuration we need to consider the azimuthal ranges coming from each one of the neighboring particles:

(12) $$\begin{array}{cc} f_{b}^{r}\left(\alpha ,\gamma \right)= & \cos^{2}\gamma \left[\theta (\gamma )\theta (\pi /2-\gamma )+\theta (\gamma -3\pi /2)\theta (2\pi -\gamma )\right]+\sin^{2}\left(\alpha /2+\gamma \right)[\theta \left(\gamma \right)\theta \left(\pi -\alpha /2-\gamma \right)\\ & +\theta \left(\gamma -2\pi +\alpha /2\right)\theta \left(2\pi -\gamma \right)]+\cos^{2}\left(\alpha +\gamma \right)\left[\theta (\gamma -\pi /2+\alpha )\theta (3\pi /2-\alpha -\gamma )\right]\\ & +\sin^{2}\left(3\alpha /2+\gamma \right)\left[\theta (\gamma -\pi +3\alpha /2)\theta (2\pi -3\alpha /2-\gamma )\right]+\sin^{2}\left(\alpha /2+\gamma \right)[\theta \left(\gamma -\pi +\alpha /2\right)\\ & \cdot \theta \left(2\pi -\alpha /2-\gamma \right)]+\sin^{2}\left(\alpha /2-\gamma \right)\left[\theta (\gamma -\pi -\alpha /2)\theta (2\pi -\gamma )+\theta (\gamma )\theta (\alpha /2-\gamma )\right] \end{array}$$

**Figure 4:**
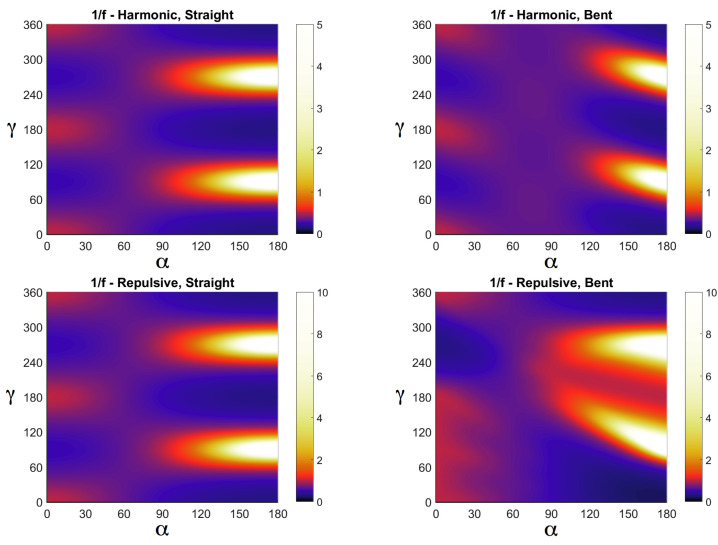
Color diagram of 1/f, as defined in the text, as a function of the angles α and γ, for harmonic and repulsive harmonic interactions and for straight- and bent-stripes configurations.

In Figure 4 we show 1/f for hamonic and repulsive harmonic interactions for straight and bent configurations as a function of the lattice deformation angle α and the azimuthal direction in space γ, over which we numerically integrate in order to get the value of the function *I* for that specific angle α, which in turn sets the entropy via Equation (9). From Figure 4 we can see, particularly for big angles α for which Δs takes its larger values (see Figure 2b), that repulsion accentuates the contribution to the function *I* for bent stripes (corresponding in Figure 4 to a wider region composed of brighter colors, i.e., white, yellow and red, for repulsive harmonic over harmonic interaction in the case of bent stripes). More in general, we can say that repulsion accentuates differences in the contribution to the partition function and thus to the free energy between symmetric and asymmetric distribution of neighboring particles.

## 3. Conclusions

We studied a 2D triangular-network model composed of particles interacting through harmonic or repulsive harmonic springs. At T=0 the ground state is degenerate and composed of straight or any set of zigzagging-stripes configurations. At T>0 we found that the stable phase is composed of straight or bent stripes depending on the harmonic or repulsive harmonic nature of particle interaction, respectively. This selection mechanism of the stable phase through the order-by-disorder effect is equivalent to that observed in the colloidal [20] and Ising [25] antiferromagnets irrespective of the dimensionality of the system. This suggests that the phase inversion of isosceles triangular networks is controlled by the attraction component of the interparticle interaction. We suggest that this is due to the fact that repulsive interactions accentuate differences in the contribution to the free energy between symmetric and asymmetric distribution of neighboring particles, as we have shown for n=1 free particle calculation.

Both the Ising antiferromagnet on a deformable triangular lattice and the 2D isosceles triangular network model at T=0 from one side, and the colloidal monolayer at $\rho =\rho_{cp}$ from the other side, have the same degeneracy and subextensive entropy $S∼\sqrt{N}$ and thus a vanishing residual entropy per particle in the thermodynamic limit. At T>0 for the triangular networks and at $\rho <\rho_{cp}$ for the colloidal monolayer this degeneracy is removed, but they can still have a residual entropy per particle for finite system size if the ergodicity is broken. Indeed, even at T>0 or $\rho <\rho_{cp}$ a system may be trapped in a local minimum of the free-energy landscape and thermal fluctuations are not large enough for a small system to overcome energy barriers. For ergodic systems the time average of observables can be computed by using ensemble averages thanks to the Birkhoff theorem [38]. From the other hand, for non-ergodic systems, the phase space is divided into disjoined sets. In this case, states can be counted either following the kinetic view [39], for which only states visited by the system at the observational time scale are taken into account, or following the Edwards approach [40], for which all possible states are considered regardless of whether they are explored or not by the system. Recently, the Edwards hypothesis has been proved to be valid at the un-jamming point [41]. In thermal ergodic systems at equilibrium, the two sampling methods give the same result. Following the Edwards approach we can conclude that an indication of the presence of residual entropy in a system is given by the ergodicity breaking (which can be checked for generic temperature or density) instead of by the degeneracy of the ground state (defined for T=0 or $\rho =\rho_{cp}$ only).

## Figures and Tables

**Table 1:** Distances between the neighboring particles and the central particle 0 in the unit cell of the straight-stripe configuration. Particle positions are graphically shown in Figure 1c.

Particles	dx	dy	${\mathrm{dr}}_{0}$
1, 0	2a	0	2a
2, 0	*a*	*b*	*c*
3, 0	−a	*b*	*c*
4, 0	−2a	0	2a
5, 0	−a	−b	*c*
6, 0	*a*	−b	*c*

**Table 2:** Distances between the neighboring particles and 0 and 1 particles in the unit cell of the maximally zigzagging-stripe configuration. Particle positions are graphically shown in Figure 1d.

Particles	dx	dy	${\mathrm{dr}}_{0}$
1, 0	2a	0	2a
4, 0	*a*	*b*	c
5, 0	$2a\left(1-8\frac{a^{2}}{c^{2}}\right)$	$4b\left(1-\frac{b^{2}}{c^{2}}\right)$	2a
6, 0	$a\left(1-4\frac{b^{2}}{c^{2}}\right)$	$b\left(3-4\frac{b^{2}}{c^{2}}\right)$	*c*
7, 0	−a	−b	*c*
8, 0	*a*	−b	*c*
2, 1	$-a\left(1-4\frac{b^{2}}{c^{2}}\right)$	$-b\left(3-4\frac{b^{2}}{c^{2}}\right)$	*c*
3, 1	*a*	*b*	*c*
4, 1	−a	*b*	*c*
8, 1	−a	−b	*c*
9, 1	$-2a\left(1-2\frac{b^{2}}{c^{2}}\right)$	$-4b\left(1-\frac{b^{2}}{c^{2}}\right)$	2a
